# Supplementary material for: piRNAs and Aubergine cooperate with Wispy poly(A) polymerase to stabilize mRNAs in the germ plasm
Source: Nat Commun. 2017 Nov 3;8:1305. doi: 10.1038/s41467-017-01431-5 (PMC5670238; doi:10.1038/s41467-017-01431-5)
Supplement: Supplementary file 1 — Supplementary Information [file 41467_2017_1431_MOESM1_ESM.pdf]

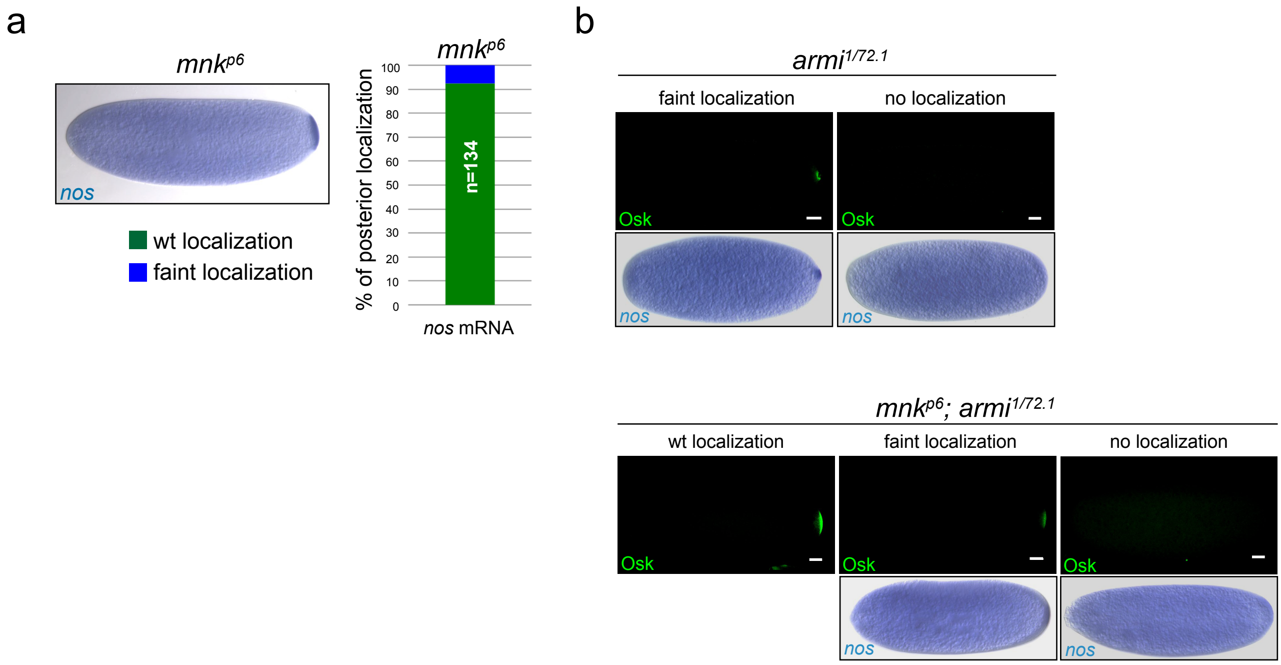

Supplementary Figure 1. Armi has a direct role in *nos* mRNA posterior localization (a) *in situ* hybridization of *nos* mRNA in 0-2 h-*mnk* mutant embryos, showing no defect in posterior localization. Quantification of *nos* mRNA posterior localization is indicated (right panel); n is the number of counted embryos. (b) Osk immunostaining and *nos* mRNA *in situ* hybridization of 0-2 h *armi* and *mnk; armi* mutant embryos. Scale bars: 30 µm.

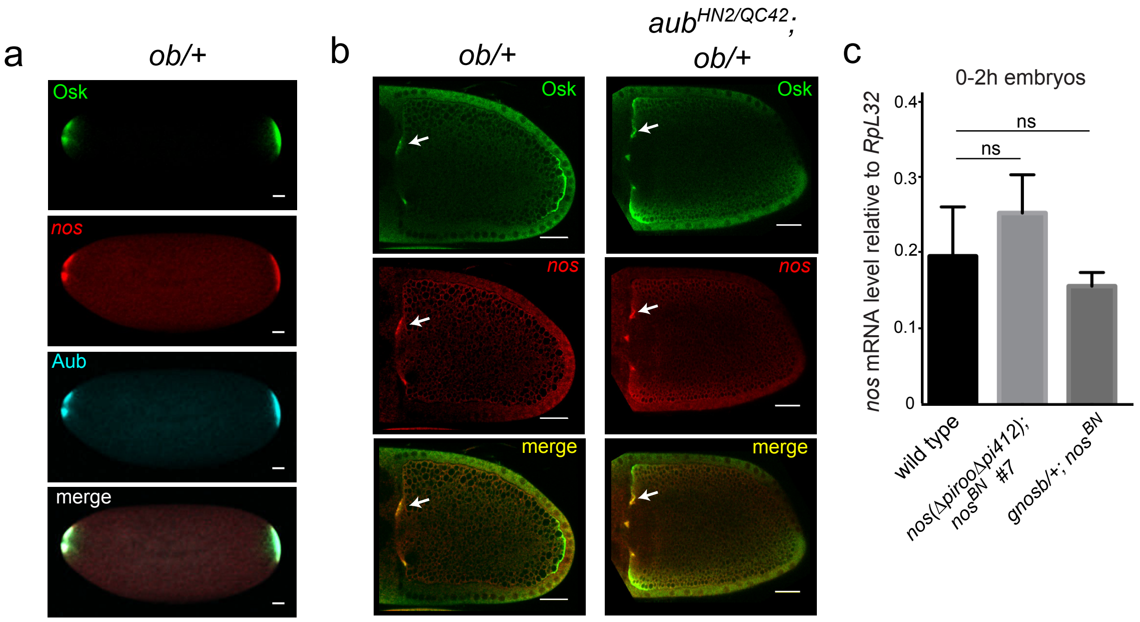

d

Targeting of Aub-interacting and non-interacting mRNAs with piRNAs using variable base pairing			
Number of mismatches (mm)	% of targeted Aub-interacting mRNAs (n=595)	% of targeted Aub-non-interacting mRNAs (n=595)	Statistical Analysis
0	40% (n=236)	30% (n=176)	2.4e-04
1	52% (n=310)	37% (n=221)	4.1e-07
2	70% (n=417)	54% (n=323)	7.5e-08
20nt seed-1mm	74% (n=443)	61% (n=365)	1.6e-06
16nt seed-0mm	87% (n=519)	80% (n=478)	9.7e-04

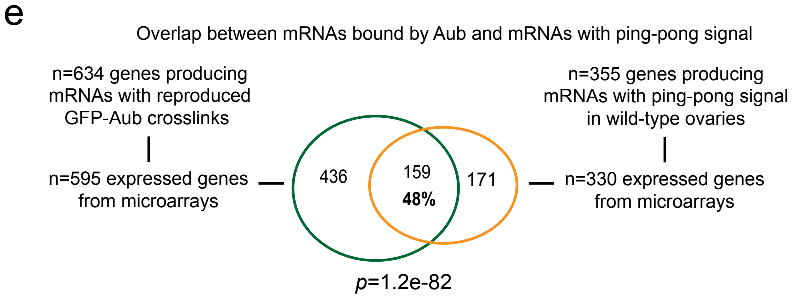

Supplementary Figure 2. Aub binding to mRNAs depends on sequence-specific base-pairing with piRNAs (a) Immuno-FISH of 0-2 h-*osk-bcd3'UTR (ob/+)* embryos with anti-Osk (green), anti-Aub (cyan) and *nos* RNA probe (red) showing the recruitment of Aub at the anterior germ plasm and its colocalization with *nos* mRNA in *ob* embryos. (b) Immuno-FISH of *ob/+* stage 10 oocytes with anti-Osk (green) and *nos* RNA probe (red), in wild-type and *aub* mutant backgrounds. White arrows show *nos* localization to the anterior germ plasm in both wild-type and *aub* mutant backgrounds. Scale bars: 30 µm in (a, b). (c) *nos* mRNA quantification using RT-qPCR in 0-2 h-embryos. Genotypes are indicated. Normalization was with *RpL32* mRNA. Means are from three to five biological replicates. The error bars represent SD. ns: non-significant using the two-tailed Student's t test. (d) Capacity of embryonic piRNAs to target Aub-bound or -unbound mRNAs with different complementarities. The percentages of targeted mRNAs among Aub-unbound mRNAs are the mean of 100 samplings of 595 mRNAs within 5743 mRNAs expressed in the embryo. The first nt of piRNAs was not considered in the base pairing. Base pairing with 16-nt or 20-nt seed indicates that nt 2-16 and 2-20 of piRNAs were considered, respectively. Statistical analysis was performed using the Fisher's exact test. (e) Venn diagram of mRNAs interacting with Aub [11] and mRNAs producing piRNAs upon targeting with a highly complementary trigger piRNA [26]. Statistical analysis was performed using the Fisher's exact test.

a c

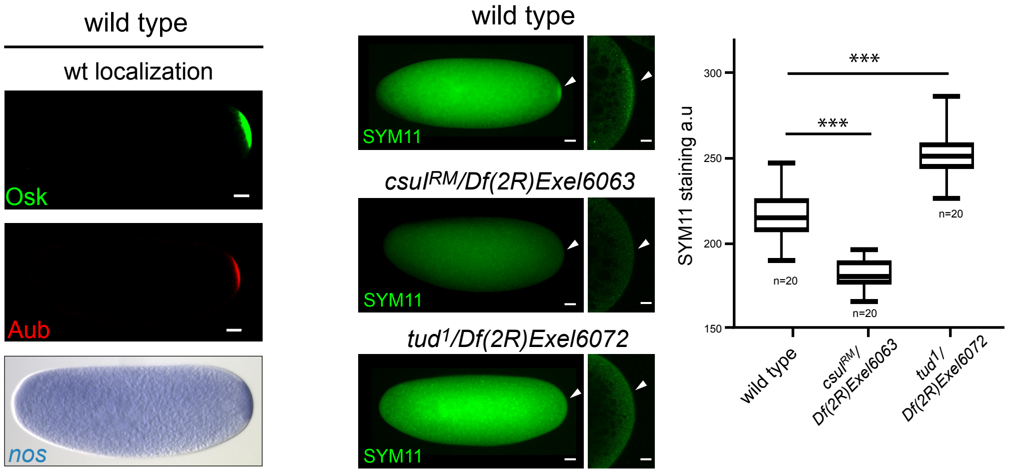

b

	wild type	*csul*^{RM}/ *Df(2R)Exel6063*	*tud*^{1}/ *Df(2R)Exel6072*
Embryonic lethality	8.1% (n=726)	48.5% (n=1152)	14.1% (n=991)

Supplementary Figure 3. Arginine dimethylation in *csul* and *tud* mutant embryos (a) Immunostaining with anti-Osk (green), anti-Aub (red), and *nos* mRNA *in situ* hybridization of 0-2 h-wild-type embryos. Scale bars: 30 µm. (b) Lethality of embryos from wild-type, *csul* and *tud* mutant females. (c) Immunostaining of wild-type, *csul* and *tud* mutant embryos with SYM11 antibody that recognizes symmetric dimethylarginines. Higher magnifications of posterior poles are shown in the right panels. Box plot showing the quantification of SYM11 staining in the soma, in arbitrary units. The central horizontal bar represents the median. *** $p < 0.001$, using the two-tailed Student's t test. 100% of embryos showed accumulation of SYM11 staining in the germ plasm in wild type (n=129) (white arrowhead), strongly reduced SYM11 staining in the whole embryo in *csul* mutant (n=116), and no accumulation in the germ plasm in *tud* mutant (n=103). Scale bars: 30 µm in left panels; 10 µm in right panels

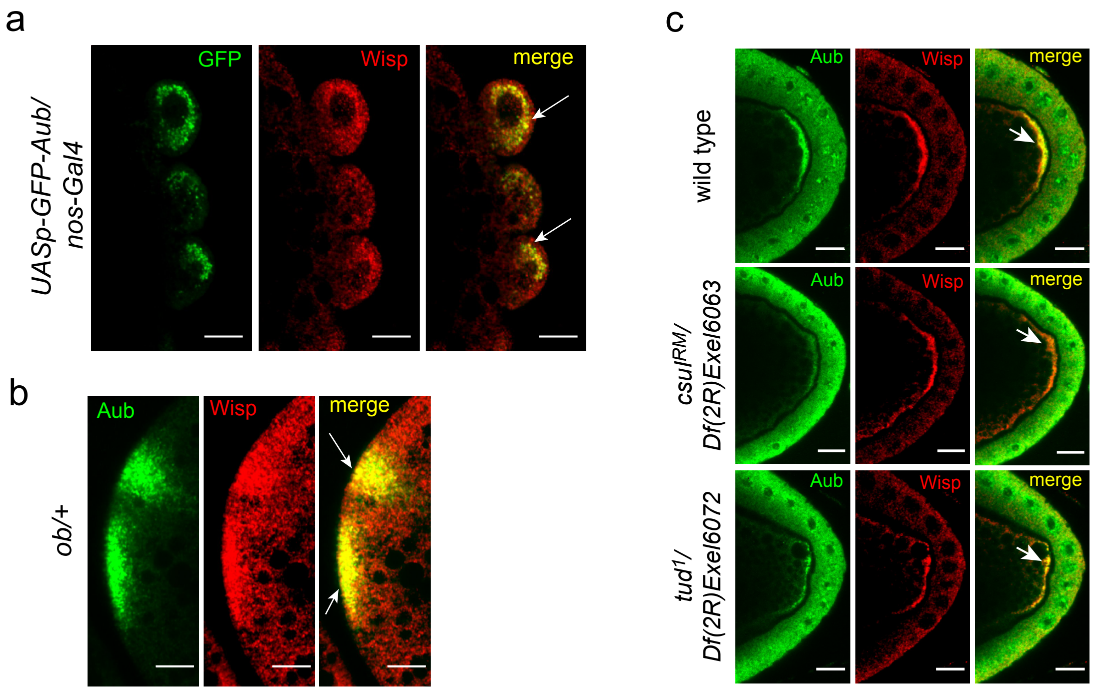

Supplementary Figure 4. Colocalization of Aub and Wisp in the germ plasm (a) Immunostaining of 0-4 h *UASp-GFP-Aub/nos-Gal4* embryos with anti-GFP (green) and anti-Wisp (red), showing Aub and Wisp partial colocalization in primordial germ cells (white arrows). (b) Immunostaining of 0-2 h *ob/+* embryos with anti-Aub (green) and anti-Wisp (red), showing the colocalization of Aub and Wisp in the anterior germ plasm (white arrows). Anterior pole is to the left. (c) Immunostaining of wild-type, *csul* and *tud* mutant stage 10 oocytes with anti-Aub (green) and anti-Wisp (red), showing the colocalization of Aub and Wisp in the germ plasm (white arrows). Scale bars: 10 µm in (a, b, c).

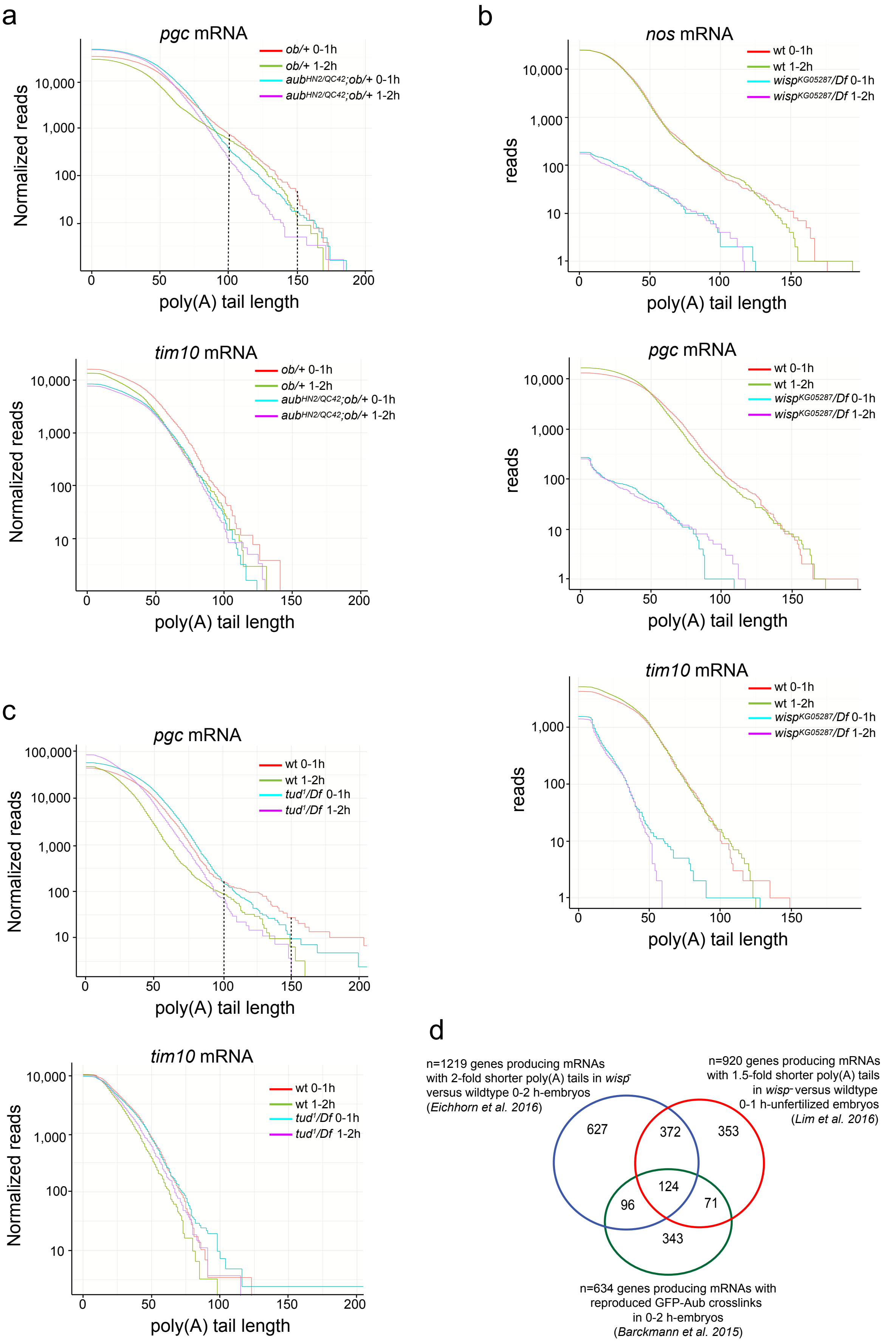

Supplementary Figure 5. mRNA polyadenylation by Wisp and Aub in the germ plasm (a) Cumulative plots of the sequenced poly(A) tails of *pgc* and *tim10* mRNAs in 0-1 h- and 1-2 h-*ob/+* embryos, in wild-type and *aub* mutant backgrounds. Each curve represents the mean of two biological replicates normalized to reads per million. The x-axis represents the number of non-templated A-bases sequenced at the end of each read; the y-axis represents the normalized number of reads on a log scale. The proportion of reads (% total) with a sequenced poly(A) tail of more than 100 bases is reduced in the *aub* mutant for *pgc* (p=0.0001) but not for *tim10* (p=0.984) by two-way ANOVA taking into consideration both time and genotype variables. (b) mPAT of *nos*, *pgc* and *tim10* mRNAs in 0-1 h- and 1-2 h-embryos from wild-type and $wisp^{KG05287}/Df(1)RA47$ females. Cumulative plots of raw sequenced read numbers. The y-axis is on a log scale. The adenylation-state and stability of *nos* and *pgc* mRNAs strongly depend on cytoplasmic polyadenylation by Wisp, while *tim10* mRNA polyadenylation and stability are less affected in *wisp* mutant. (c) Cumulative plots of the sequenced poly(A) tails of *pgc* and *tim10* mRNAs in wild-type and *tud* mutant embryos. Each curve represents the mean of two biological replicates normalized to reads per million. x- and y-axes as in (a). (d) Venn diagram of Aub-bound mRNAs and Wisp target mRNAs. 496 mRNAs were identified as Wisp targets in both studies by Eichhorn et al. [33] and Lim et al. [34]; among those, 124 (25%) were bound by Aub.

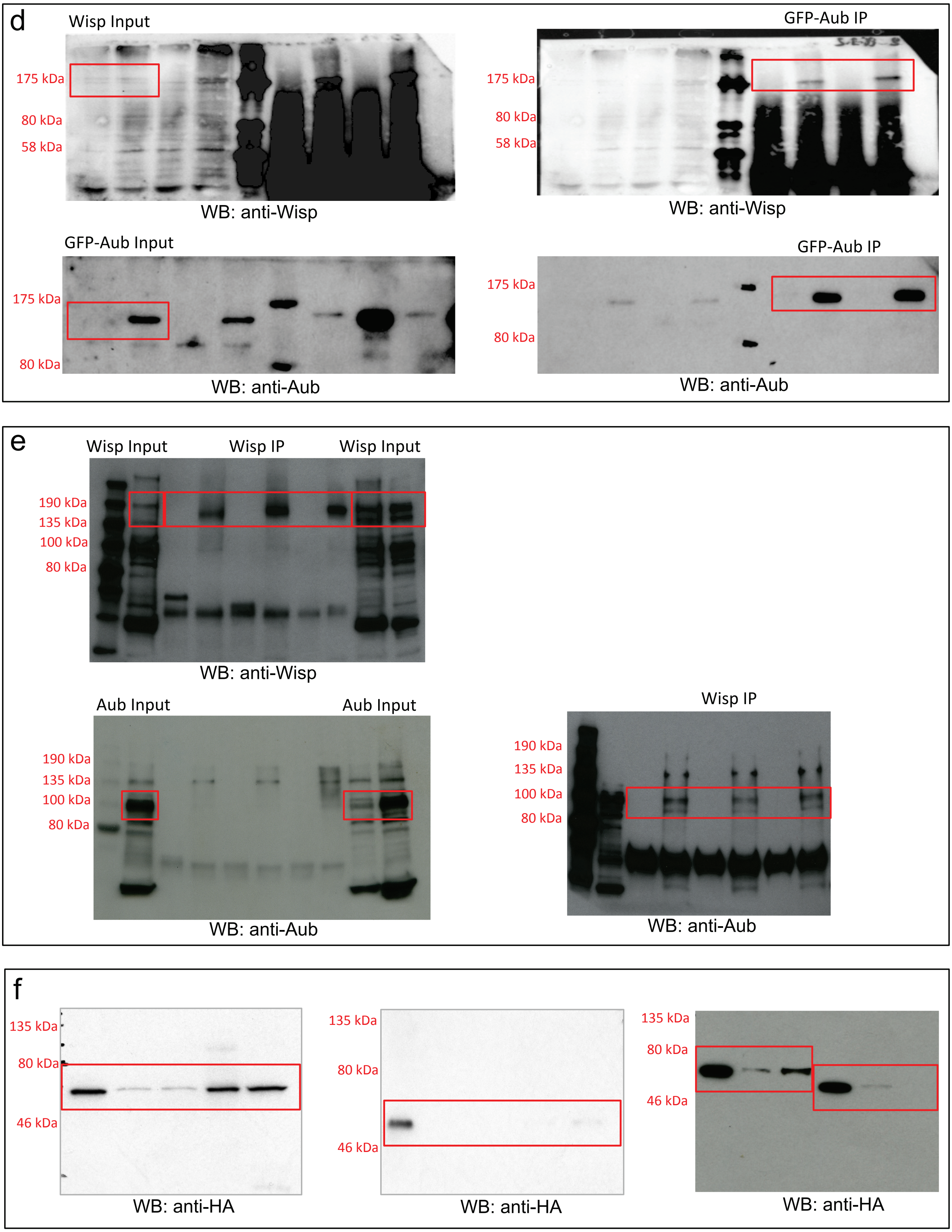

Supplementary Figure 6. Complete blots relative to Figure 4
